# Comprehensive analysis of prognostic value, relationship to cell cycle, immune infiltration and m6A modification of ZSCAN20 in hepatocellular carcinoma

**DOI:** 10.18632/aging.204312

**Published:** 2022-12-03

**Authors:** Fang Jiayu, Yike Jiang, Xuanrui Zhou, Minqin Zhou, Jingying Pan, Yun Ke, Jing Zhen, Da Huang, Weifan Jiang

**Affiliations:** 1 Second Affiliated Hospital of Nanchang University, Nanchang, China; 2 Second College of Clinical Medicine, Nanchang University, Nanchang, China; 3 Department of Thyroid Surgery, Second Affiliated Hospital of Nanchang University, Nanchang, China; 4 Department of Urology, Second Affiliated Hospital of Nanchang University, Nanchang, China

**Keywords:** hepatocellular carcinoma, ZSCAN20, biomarker, prognosis, immune infiltrates

## Abstract

Hepatocellular carcinoma (HCC) is a common tumor across the globe with a high mortality rate. ZSCAN20 is a ZNF transcription factor, a key determinant of gene expression. Nonetheless, the mechanism of ZSCAN20 as a potential clinical biomarker and therapeutic target for HCC is not understood. Here, TIMER, TCGA, ICGC databases and immunohistochemical (IHC) and Western Blot found ZSCAN20 mRNA and protein levels were upregulated. Additionally, Kaplan-Meier Plotter, GEPIA and TCGA databases showed high ZSCAN20 expression was related to the short survival time of HCC patients. Multivariate Cox analysis exposed that ZSCAN20 can act as an independent prognostic factor. We observed methylation level of ZSCAN20 was associated with the clinicopathological characteristics and prognosis of HCC patients through UALCAN. Furthermore, enrichment examination exposed functional association between ZSCAN20 and cell cycle, immune infiltration. Functional experiments showed that interference with ZSCAN20 significantly reduced the invasion, migration and proliferation abilities of HCC cells. An immune infiltration analysis showed that ZSCAN20 was associated with immune cells, particularly T cells. The expression of ZSCAN20 was correlated with poor prognosis in the Regulatory T-cell. And Real-Time RT-PCR analysis found interference with ZSCAN20 significantly reduced the expression of some chemokines. Finally, the TCGA and ICGC data analysis suggested that the ZSCAN20 expression was greatly related to m6A modifier related genes. In conclusion, ZSCAN20 can serve as a prognostic biomarker for HCC and provide clues about cell cycle, immune infiltration, and m6A modification.

## INTRODUCTION

Hepatocellular carcinoma (HCC) accounts for 90% of primary liver cancer [1]. HCC is the second reason to lead cancer-related demise all over the world, because of the accelerated progress of HCC [2–4]. Also, many patients have reached the advanced stage when they are diagnosed, bringing about a poor prognosis for HCC patients, with a 5-year survival rate of only 18% [5]. If patients get an early treatment, it will extremely better the survival rate of patients. In view of the discovery of HCC biomarkers, the diagnosis of HCC has made accelerated progress. For example, Alpha Fetoprotein (AFP) check is utilized in the early diagnosis of HCC, but it still has a certain misdiagnosis rate [6]. Early patient with HCC can be treated by surgical resection and ablation, but the tumor recurrence rate is still high, which is not so ideal [7]. Meanwhile, more and more evidence reveals that immunotherapy including immune checkpoint blockade can become a likely optional therapy for HCC [8–10]. However, useful immunotherapy targets for HCC are still deficient. Therefore, it is still crucial to find some more effective HCC biomarkers and therapeutic targets.

The ZSCAN family belongs to zinc finger transcription factors. As the family that contains the most transcription factors [11], zinc finger transcription factors are characterized by finger-like DNA binding domains, which require one or more zinc ions to stabilize the structure and are used in many biological processes and play an important role [12]. The human ZSCAN transcription factor family has 54 identified members. As a member of this family, ZSCAN20 has a chromosome located at 1p35.1. ZSCAN transcription factors may be involved in angiogenesis, cell apoptosis, cell differentiation, cell migration and invasion, cell proliferation, stem cell characteristics, and chemotherapy sensitivity. Increasingly, studies are detecting that abnormal ZSCAN transcription factor expression in liver cancer is linked to significant biological functions. In HCC, ZSCAN3 directly binds to the promoter of β-catenin to activate its transcription and promote cell proliferation [13], and ZSCAN33 can inhibit HCC cell growth, migration, and invasion [14]. In addition, ZSCAN35 is directly related to carcinoembryonic antigen and takes an active part in the growth of HCC metastasis; ZSCAN35 may promote liver metastasis [15]. Furthermore, recent studies have found that ZSCAN20 may become a new target for tumor treatment. The article reports that ZSCAN20 is highly expressed in low-grade gliomas and can be used as a part of the model to predict the prognosis of low-grade gliomas. Nevertheless, the effect and mechanism of ZSCAN20 in HCC haven’t been reported, and its relationship with the prognosis is still unclear.

There are few studies on ZSCAN20, and the mechanism of ZSCAN20’s role in cancer has not yet been elucidated. As a consequence, here we explore the specific mechanism of ZSCAN20 as a prognosis and potential therapeutic target for HCC patients.

## MATERIALS AND METHODS

### Patients and tumor specimens

We gathered 40 pairs of paraffin-embedded HCC and corresponding normal tissues from 40 patient samples from the Second Affiliated Hospital of Nanchang University from January 2018 to January 2021. The above patients did not receive adjuvant chemotherapy. Entire patients endorsed a knowledgeable consent form. Also, this research was accepted by the Ethics Committee of the Second Affiliated Hospital of Nanchang University.

### Data collection

The TCGA database (https://cancergenome.nih.gov) [16] includes sequencing and clinicopathological data from more than 32 cancer patients. We evaluated the ZSCAN20 expression pattern according to various cancer research from the LIHC data of the Cancer Genome Atlas (TCGA), including 377 cancer samples and 50 normal samples. The main goal of ICGC is to comprehensively clarify the genomic changes in many cancers that contribute to the global burden of human disease [17]. ICGC collects tumor data of 50 different cancer types. The used file type was [LINC-JP] Liver Cancer-NCC, JP data set, which includes 243 tumor samples and 260 clinical samples.

### TIMER database analysis

TIMER (https://cistrome.shinyapps.io/timer/) [18–21] is a broad capability library for analyzing the immune infiltration of different cancer types systematically. It concludes 1,000 samples of 32 cancer types. The degree of immune cell infiltration in tumor tissues can be appraised from RNA-seq expression profile data, reflecting the correlation hidden in cancer and immune cells. We analyzed the expression of ZSCAN20 in various cancer types and the interaction between ZSCAN20 expression and immune infiltration, along with conventional immune cells. Then, we used the SCAN module of TIMER database to analyze the relationship between ZSCAN20 CNV and immune cell infiltration. In addition, the TIMER database was also utilized to study the interaction hidden in ZSCAN20 expression and various immune gene marker sets of immune cells.

### Cell culture

Transfection Human HCC cell lines LM3 were maintained with 5% CO2 at 37° C in DMEM (HyClone Germany) with 10% fetal bovine serum (GIBCO USA).

### Immunohistochemistry

The HCC tissues and paired adjacent tissues fixed with 10% formalin and implanted in paraffin were cut into 4um thick sections. After deparaffinization, rehydration, and microwave heating in sodium citrate buffer (10 mmol/L, pH 6.0) for 25 minutes to restore the antigen, the sections were sealed with goat serum for 30 minutes. Then, the sections were incubated overnight with anti-ZSCAN20 polyclonal antibodies (ZSCAN20-LifeSpan BioSciences-Catalog number: C660829) at 4° C. Next, the HRP conjugated second antibody (booster) was allowed to stand at room temperature for 2 hours. Subsequently, immunostaining was performed using a two-step method. Pathologists who were unaware of the clinical parameters assessed the staining intensity and the percentage of positive cells semi-quantitatively [22].

### Western blot

For the preparation mean of Western Blot total protein extract, saw [23]. In general, protein extraction on ice using RIPA buffer (Beyotime, Shanghai, China) which was a mixture of protease and inhibitor (Thermo Fisher Scientific, New York, USA). After centrifugation, the protein concentration was observed using the BCA Protein Detection Kit (Thermo Scientific, Waltham, MA, USA), and an equal amount of protein was isolated on SDS-PAGE by electrophoresis, which was transferred to a polyvinylidene fluoride (PVDF) membrane. Then incubate overnight at 4° C applying the primary antibody, followed by washing the closed membrane 3 times in TBST, while incubating with the secondary antibody for 1h at room temperature. After the above process was accomplished, the protein expression was checked employing electro-chemiluminescence (ECL) assay.

### Quantitative real-time PCR

Real-time PCR was performed on fresh frozen samples. Total RNA was isolated from tissues using the TRIzol reagent (Thermo Fisher Scientific). Then, according to standard methods, qRTPCR analysis was performed to observe the expression level of ZSCAN20. Primers for ZSCAN20 from 5’ to 3’: (F), (R). Primers for β-actin from 5’ to 3’: (F), (R).

### HPA analysis

HPA (https://www.Proteinatlas.org/) uses antibody methods for immunostaining of tissues and cell lines, along with contrasting expression analysis of proteins in normal and tumor tissues [24]. In this research, the expression of ZSCAN20 was disclosed by using the protein expression module in the HPA database, and the immunohistochemical outcomes of ZSCAN20 in tumor tissues and normal tissues were concluded.

### UALCAN analysis

UALCAN (http://ualcan.path.uab.edu/) is an interactive evaluation and excavation web on the Internet, which can be used to analyze the relationship between tumor and normal specimens and the relative expression of genes with diverse clinicopathological characteristics [25, 26]. UALCAN was used to evaluate the differential expression of ZSCAN20 in HCC tissues and normal tissues. The research further investigated various clinicopathological characteristics of ZSCAN20 and the methylation of ZSCAN20 promoter.

### MethSurv database analysis

MethSurv (https://biit.cs.ut.ee/methsurv/) is an open online instrument, which can evaluate the prognostic value of CpG methylation data. The methylation data comes from The Cancer Genome Atlas (TCGA), which can further provide overall survival (OS) of DNA methylation levels. We could use this database to analyze the DNA methylation data of ZSCAN20 and evaluate the prognostic value of a single CpG site in HCC patients [27, 28].

### GEPIA database analysis

Gene expression profiling transactional estimation (GEPIA) (http://gepia.cancer-pku.cn/) is an interactional website application consisting of thousands of tumor and normal tissue sample data, which can be used to visualize clinicopathological characteristics. The tumor data comes from the TCGA database [24]. We used GEPIA to parallel tumors with normal tissues and analyzed related prognosis in this research. In addition, the relationship between ZSCAN20 and CD274, CTLA4, PDCD1 was firmed in the “correlation analysis”. The overall survival of the m6A-related genes HNRNPA2B1, RBMX, YTHDC1 and YTHDF2 in LIHC were also generated through this locus.

### TISIDB analysis

Tumor and immune system interaction database (TISIDB) (http://cis.hku.hk/TISIDB) is a portal site, which can be used to evaluate how the tumor interacts with the immune system, and it integrates various heterogeneous data types [29]. Using the TISIDB database, we investigated the features of ZSCAN20 and its function in tumor immune interaction. The “Chemokine” module was used to analyze spearman correlations between ZSCAN20 and chemokines across LIHC.

### Kaplan-Meier plotter database analysis

The Kaplan-Meier database (http://kmplot.com/analysis/) can be used to appraise the effect of genes on the survival of cancer tissue samples [30, 31]. We used 364 LIHC samples to evaluate the interaction hidden in ZSCAN20 expression and overall survival (OS), relapse-free survival (RFS), and disease-specific survival (DSS). We also explored the difference in LIHC patients’ survival under different immune cell subtypes. A 95% confidence interval and log-rank p < 0.05 was studied analytically.

### LinkedOmics database analysis

The website named LinkedOmics database has been utilized to analyze 32 TCGA cancer-related data sets (http://www.linkedomics.org/login.php) [32–35]. Our team utilized the LinkFinder module of LinkedOmics to research the differently expressed genes connected with ZSCAN20 in TCGA LIHC (n = 515). Perform statistical analysis on the conclusions and display them in volcano maps, heat maps, and scatter plots. Gene set enrichment analysis (GSEA) has been utilized for LinkedOmics functional modules to conduct gene ontology (GO) analysis and KEGG pathway analysis. False discovery rate (FDR) less than 0.01 is a noteworthy expression, P-values less than 0.05 is a significantly related gene.

### GSEA software analysis

GSEA is an analysis way that confirms if a previously confirmed set of genes reveals a statistically significant and consistent difference amongst two phenotypes [34, 36]. The R package clusterProfiler (4.1.0) [35] has been utilized for performing GSEA to clarify the noteworthy functional and pathway differences amongst ZSCAN20 high expression group and ZSCAN20 low expression group. We performed 1,000 analyses of the genome arrangement and used the expression level of ZSCAN20 mRNA as a phenotypic marker. After running GSEA, P < 0.05 and FDR < 0.25 are both meaningful.

### Protein-protein interaction (PPI) network analysis

The online inquire implement (STRING, https://string-db.org) for finding interacting genes which are utilized for PPI network construction and pivot gene screening [37, 38]. Besides, with the purpose of exploring the correlation amongst the top 500, our team used STRING database for analysis, medium confidence=0.4 for screening, and visualization using Cytoscape software. To search cluster sub-networks, the Cytoscape Molecular Complex Detection (MCODE) plug-in has been utilized [39]. The acquiescent parameters are listed below: degree cutoff = 5, node score cutoff = 0.2, k-core = 9, maximum depth = 100.

### Transwell assay

The stably transfected cells 48 hours after seeding were subjected to Transwell invasion assay to evaluate the invasive ability of ZSCAN20. For migration, 6 x 10^4^ cells were plated in the upper chamber with serum-free medium. For invasion, 1 x 10^5^ cells were placed in Matrigel-coated chambers (BD Biosciences). Subsequently, after incubation at 37° C for 24 hours (to check migration) or 48 hours (to check invasion), Transwell inserts and invasive cells were fixed in methanol for 30 minutes, stained with 0.1% crystal violet, using a CCD with DP70. The light microscope of the system (Olympus Corp.) counted the number of cells in five random fields.

### Colony formation assay

For the colony formation assay, 0.6×10^3^ cells were seeded in 6-well plates. Once the appropriate colony size had formed, cells were fixed with 4% paraformaldehyde for 30 minutes, and after that stained by 1.0% crystal violet for 30 mins until formed visible clones. Colonies were stained with Giemsa and counted in 10 different fields [40].

### Statistical analysis

R software (version 3.6.3/4.1.2) was utilized to complete all statistical analyses amongst our research. The “limma” and “beeswarm” packages of “R” and the rank-sum test were utilized to detect the difference of ZSCAN20 expressions between LIHC samples and normal samples. We explored the association between ZSCAN20 and clinicopathological features through logistic regression. Then, we used the Kaplan-Meier method to generate survival curves, and performed univariate and multivariate Cox regression analysis in R. Our team established time-dependent receiver operating characteristic curves (ROC) for comparing disparate survival factors. The graphics part below the curve (AUC) was obtained to utilize the R package “pROC”. The p value < 0.05 was affirmed statistically significant. In order to study the discrepant expression of m6A-related genes amongst ZSCAN20 high expression group and ZSCAN20 low expression group, we utilized T test analysis.

## RESULTS

### ZSCSN20 expression is increased in HCC patients

**Figure 1:**
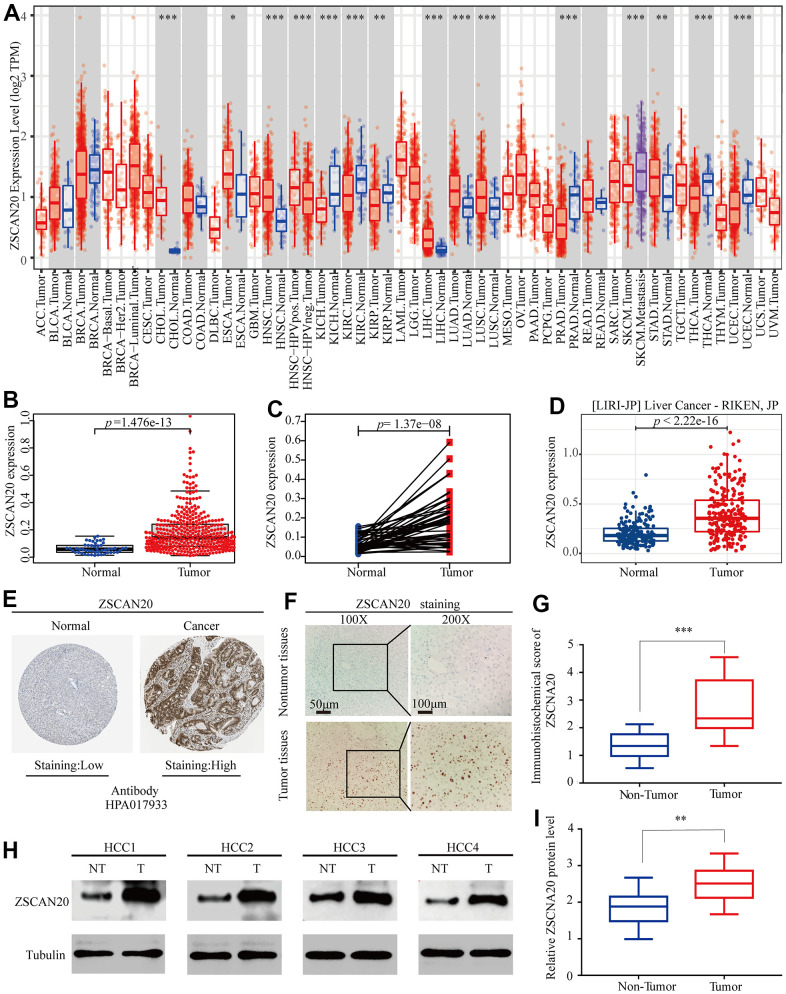
**Expression of ZSCAN20 in HCC.** (**A**) The expression level of ZSCAN20 in different types of tumor tissues and normal tissues in the TIMER database (P < 0.05). (**B**) Expression levels of ZSCAN20 were higher than corresponding normal tissues in LIHC samples (TCGA-LIHC) (p=1.476e-13). (**C**) ZSCAN20 expression in 50 paired LIHC tissues and corresponding adjacent non-tumor tissues (TCGA-LIHC) (p=1.37e-08). (**D**) Expression levels of ZSCAN20 were higher than corresponding normal tissues in LIHC samples by using ICGC-LIRI-JP liver datasets. (**E**) ZSCAN20 protein expression in normal and LIHC tissues (HPA). (**F**) Typical images of immunohistochemistry (IHC) in 35 pairs of LIHC tissues showing the protein expression of ZSCAN20 in LIHC and adjacent non-tumor tissues. (**G**) Diagram of ZSCAN20 staining score in IHC staining. (**H**) The protein expression of ZSCAN20 in tumor tissues and adjacent normal tissues was detected by Western Blot, and the coloring depth represented the level of protein expression. (**I**) Quantification of ZSCAN20 protein expression based on western blot.

The difference between ZSCAN20 expression in tumor and normal tissues was detected. Initially, we used the TCGA, TIMER and ICGC databases to assess the difference between ZSCAN20 expression in specific tumor types. We discovered that the expression of ZSCAN20 was up-regulated in LIHC and many other tumors based on the TIMER database (Figure 1A). In addition, compared with 50 adjacent normal tissue samples, ZSCAN20 in 377 LIHC samples was clearly up-regulated (Figure 1B). Subsequently, compared with adjacent normal samples, the expression of ZSCAN20 in 58 pairs of LIHC tumor samples was significantly increased (Figure 1C). The data set LIRI-JP (liver cancer-RIKEN, JP) we downloaded from the ICGC database consistently showed that the expression of LIHC in tumor samples increased (Figure 1D). In addition, we also used HPA database to detect that ZSCAN20 protein expression in tumor tissues was remarkably higher than normal tissues in the liver of patients with HCC (Figure 1E). Further studying ZSCAN20 protein expression in HCC tissues by IHC staining, we discovered that ZSCAN20 protein expression in HCC tissues was considerably higher than adjacent tissues (Figure 1F). For the aim of examine the expression level of ZSCAN20 in HCC tissues, we performed IHC staining and examined 40 pairs tumor tissues of paraffin embedded archived HCC and corresponding 40 pairs paracancerous tissues. Our data indicated that ZSCAN20 was significantly overexpressed in HCC tissues (Figure 1G). Eventually, we did a set of Western Blot experiments. As presented in Figure 1H, we dug out that in comparison with neighboring normal tissues, ZSCAN20 was more expressed in HCC tissues. Consistently, profit from qRT-PCR, our result demonstrated that the mRNA expression of ZSCAN20 was dramatically upregulated in HCC specimens in comparation with the level in adjacent normal tissues (Figure 1I). These results indicate that ZSCAN20 expression is up-regulated in HCC, and ZSCAN20 may play a crucial regulatory part in the development of HCC.

### ZSCAN20 clinical parameters of HCC patients

**Figure 2:**
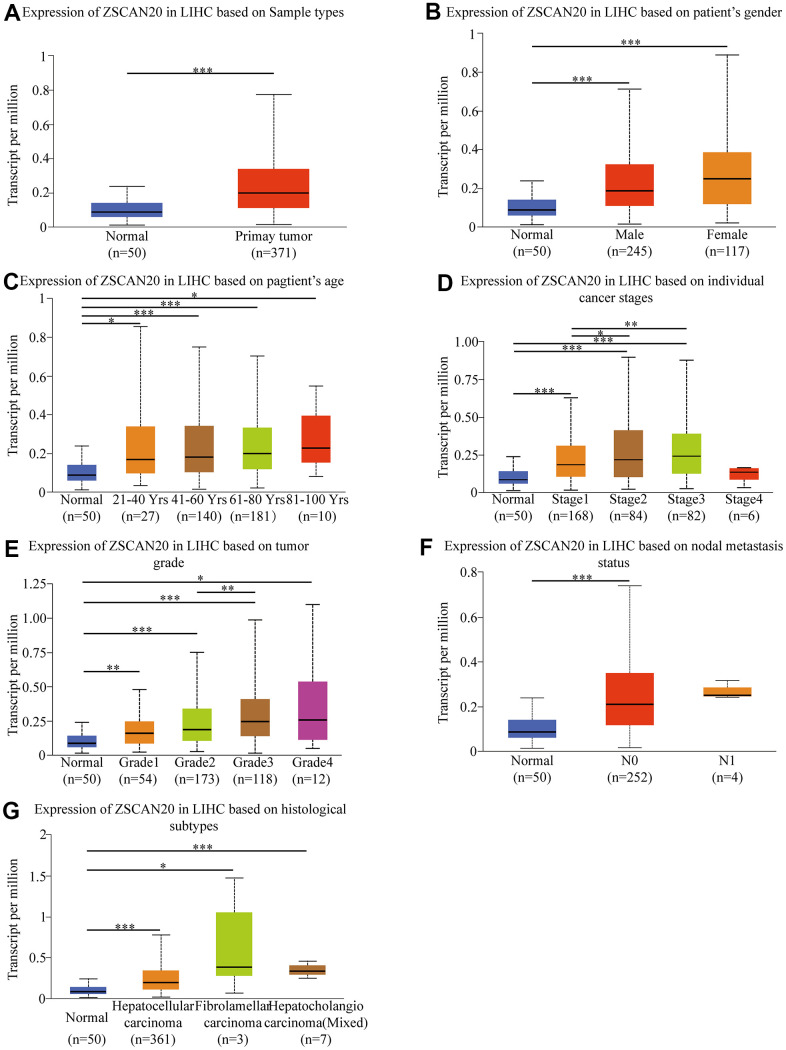
**Box-plots exploring the relationship between ZSCAN20 expression and clinicopathological characteristics (UALCAN).** Increased ZSCAN20 expression was significantly with (**A**) sample type, (**B**) gender, (**C**) age, (**D**) cancer stage, (**E**) tumor grade, (**F**) nodal metastasis status. (**G**) histological subtype *P < 0.05; **P < 0.01; ***P < 0.001.

Because we are still uncertain about the role of ZSCAN20 in HCC, studying the correlation between ZSCAN20 expression and clinicopathological features would help us to uncover ZSCAN20’s function in the development of HCC. Consequently, we used the UALCAN online tool to investigate the expression of ZSCAN20 in the patient group based on different clinical parameters. The results showed that ZSCAN20 expressed significantly different in various HCC samples, gender, age, cancer stages, tumor grade, nodal metastasis status, histological subtypes (Figure 2A–2G). We used logistic regression analysis to further study ZSCAN20 expression connected with clinicopathological variables. The results indicated the high expression of ZSCAN20 was obviously associated with grade, stage and tumor size (Supplementary Table 1). All results show that ZSCAN20 expression has a close connection with clinicopathological factors such as tumor progression and metastasis.

### High ZSCAN20 expression is associated with poor prognosis of HCC

**Figure 3:**
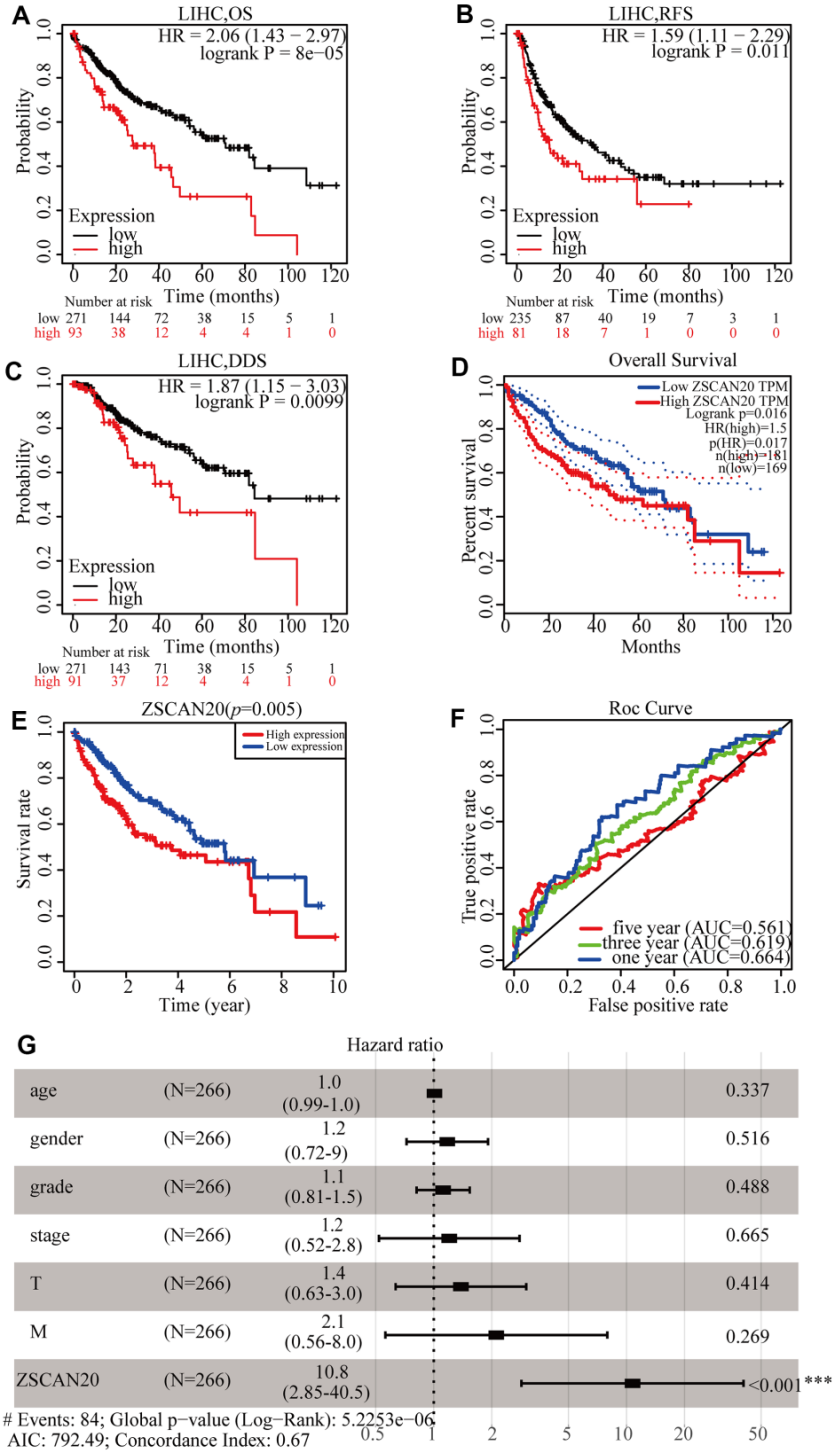
**ZSCAN20 expression was associated with poor survival in HCC patients.** (**A**–**C**) Survival curves using the Kaplan-Meier plotter are shown for OS, RFS, and DSS. (**D**) Survival curves using GEPIA is shown for OS. (**E**) Associations with overall survival and the expression of ZSCAN20 in TCGA patients. (**F**) ROC, receiver operating characteristic; ROC curve sensitivity and specificity analysis of LIHC, AUC, area under the curve. (**G**) A forest plot showed the correlation between ZSCAN20 expression and clinicopathological parameters in LIHC patients. *P < 0.05; **P < 0.01; ***P < 0.001. HR, hazard ratio; CI, confidence interval; T, tumor; N, node, M, metastasis; OS, overall survival; AIC, Akaike information criterion.

To understand the prognostic value of the expression of ZSCAN20 in HCC better, the Kaplan-Meier Plotter tool was used to detect the prognosis of ZSCAN20. Kaplan-Meier curve and log-rank test analysis showed that elevated ZSCAN20 mRNA levels correlated with overall survival (OS)(Figure 3A), relapse-free survival (RFS) (Figure 3B), and disease-specific survival (DSS) (Figure 3C). The consequence demonstrated that high ZSCAN20 expression corresponded to undesirable prognosis (OS, RFS, and DSS) in HCC patients (p < 0.05) (Figure 3A–3C). We used the GEPIA and TCGA database to further verify the conjecture, and the results were consistent with the Kaplan-Meier Plotter tool (Figure 3D, 3E). Subsequently, the prediction accuracy of survival curve was determined by the construction of ROC curve. The graphics part below the ROC curve (AUC) for 1, 3, and 5 years were severally 0.664, 0.619, and 0.561. The result indicated favorable prediction accuracy (Figure 3F). In order to determine whether ZSCAN20 is an independent prognostic factor for HCC sufferers, we conducted the Cox regression analysis. Univariate Cox regression analysis showed that grade, T stage, and ZSCAN20 were noteworthy related to OS in patients with HCC. Multivariate Cox regression analysis revealed that ZSCAN20 was an independent factor affecting the prognosis in HCC (Table 1), also did forest plot (Figure 3G). The above results indicate that ZSCAN20 can be deemed to be an independent prognostic factor for the survival of HCC sufferers, and high expression of ZSCAN20 corresponds with the poor prognosis of HCC patients.

**Table 1:** Univariate and multivariate COX regression analysis of factors associated with OS in HCC patients.

**Variable**	**Univariate analysis**			**Multivariate analysis**		
	**HR**	**95%CI**	**P-value**	**HR**	**95%CI**	**P-value**
**age**	1.007	0.990-1.024	0.441	1.009	0.991-1.026	0.337
**gender**	0.839	0.536-1.314	0.443	1.175	0.722-1.912	0.516
**grade**	1.073	0.795-1.449	0.645	1.119	0.814-1.538	0.488
**stage**	1.809	1.426-2.294	**< 0.001**	1.204	0.520-2.790	0.665
**T**	1.767	1.415-2.207	**< 0.001**	1.384	0.635-3.018	0.414
**M**	3.892	1.223-12.386	0.021	2.112	0.561-7.951	0.269
**ZSCAN20**	16.883	4.940-57.699	**< 0.001**	10.752	2.853-40.530	**< 0.001**

OS, overall survival; HR, hazard ratio; CI, confidence interval; T, tumor; N, node; M, metastasis; Bold values indicate P-values<0.05.

### DNA methylation level of ZSCAN20 in HCC patients

**Figure 4:**
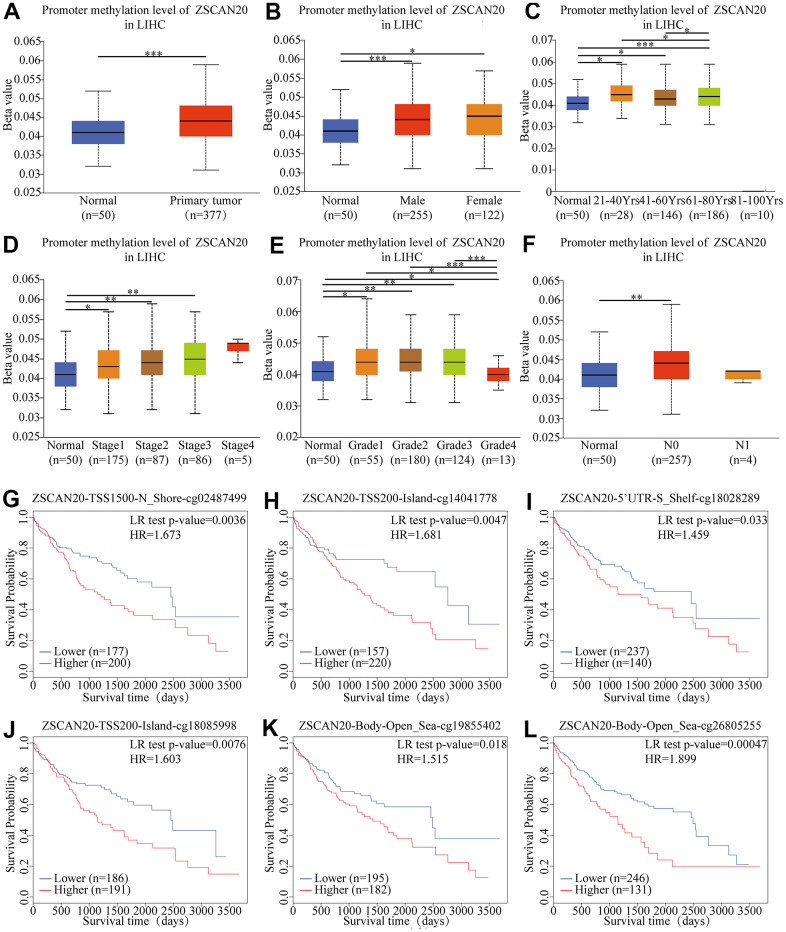
**Correlation between ZSCAN20 promoter methylation level and prognostic value of DNA methylation in HCC.** (**A**) normal vs primary tumor, (**B**) gender, (**C**) age, (**D**) cancer stage, (**E**) tumor grade, (**F**) lymph node metastasis status; *P < 0.05; **P < 0.01;***P < 0.001. High methylation level of cg02487499 (**G**), cg14041778 (**H**), cg18028289 (**I**), cg18085998 (**J**), cg19855402 (**K**) and cg26805255 (**L**) correlated with worse OS.

According to the report, DNA methylation is a chemical modification of DNA. For the purpose of studying the expression level of ZSCAN20 methylation in HCC, we based on the methylation characteristics of ZSCAN20 promoter in HCC patients and then UALCAN database was utilized to verify the methylation level of ZSCAN20 promoter in HCC. We found that the ZSCAN20 promoter in HCC was hypermethylated compared with normal tissues (Figure 4A). At the same time, it was worth noting that the ZSCAN20 promoter methylation profile based on gender, age, cancer stage, tumor grade and nodal metastasis status showed significant differences (all P < 0.01) (Figure 4B–4F). In addition, we investigated the relationship between ZSCAN20 methylation level and OS. MethSurv analysis showed that the 6 CpG sites stood on CpG islands have an undesirable prognosis, containing cg02487499, cg14041778, cg18028289, cg18085998, cg19855402, and cg26805255 (Figure 4G–4L). These results testify that the methylation of ZSCAN20 promoter is bound up with HCC’s occurrence and development.

### Co-expression networks, GO and KEGG enrichment analysis reveal pathways Of ZSCAN20 in HCC

**Figure 5:**
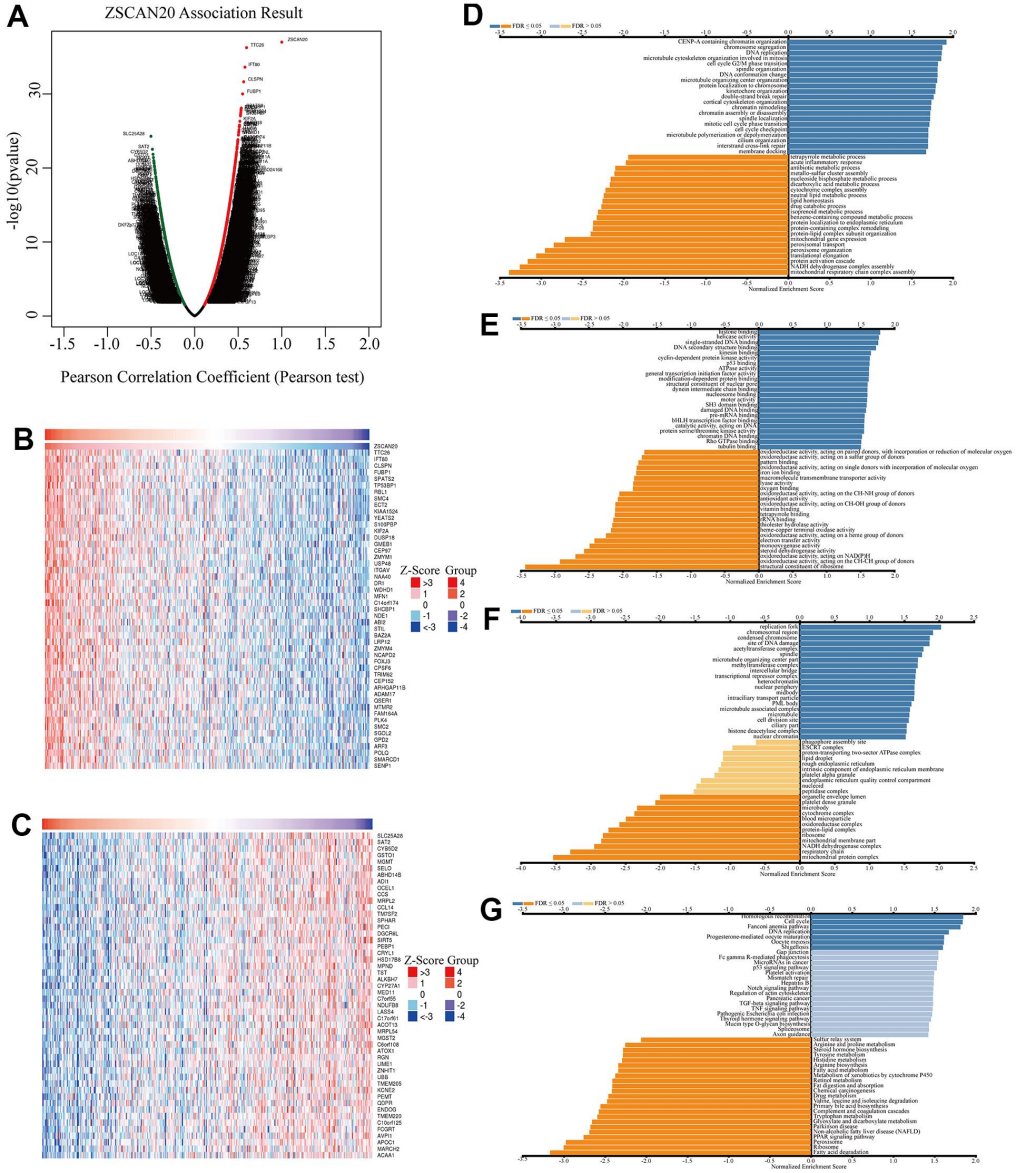
**Co-expression genes of ZSCAN20 in HCC.** (**A**) Volcano plot of genes highly correlated with ZSCAN20 identified by the Pearson test in LIHC. Red and green dots represent genes significantly positively and negatively correlated with ZSCAN20, respectively. Heatmaps of the top 50 genes (**B**) positively and (**C**) negatively correlated with ZSCAN20. (**D**–**G**) Significantly enriched GO and KEGG pathways of ZSCAN20. GO: Gene Ontology; KEGG: Kyoto Encyclopedia of Genes and Genomes.

For the sake of better exploring the biological functions of ZSCAN20 in HCC, “LinkFinder” in LinkedOmics was utilized to analyze 371 LIHC patients’ mRNA samples in TCGA. In accordance with the volcano map, there were 6713 truly positively correlated genes (red dots), and 2689 truly negatively correlated genes (green dots) (FDR < 0.01, p-value < 0.05) (Figure 5A). In addition, the first 50 negatively significant and positively significant related genes are shown in the heat maps (Figure 5B, 5C). We further explored the potential functional pathways through Linkedomics. The GO annotation showed that the co-expressed genes of ZSCAN20 mainly in chromosome separation, cell cycle G2/M phase transition, p53 binding etc. (Figure 5D–5F). KEGG pathway analysis showed that it was rich in homologous recombination, cell cycle, Fanconi anemia pathway, DNA replication, etc. (Figure 5G).

**Figure 6:**
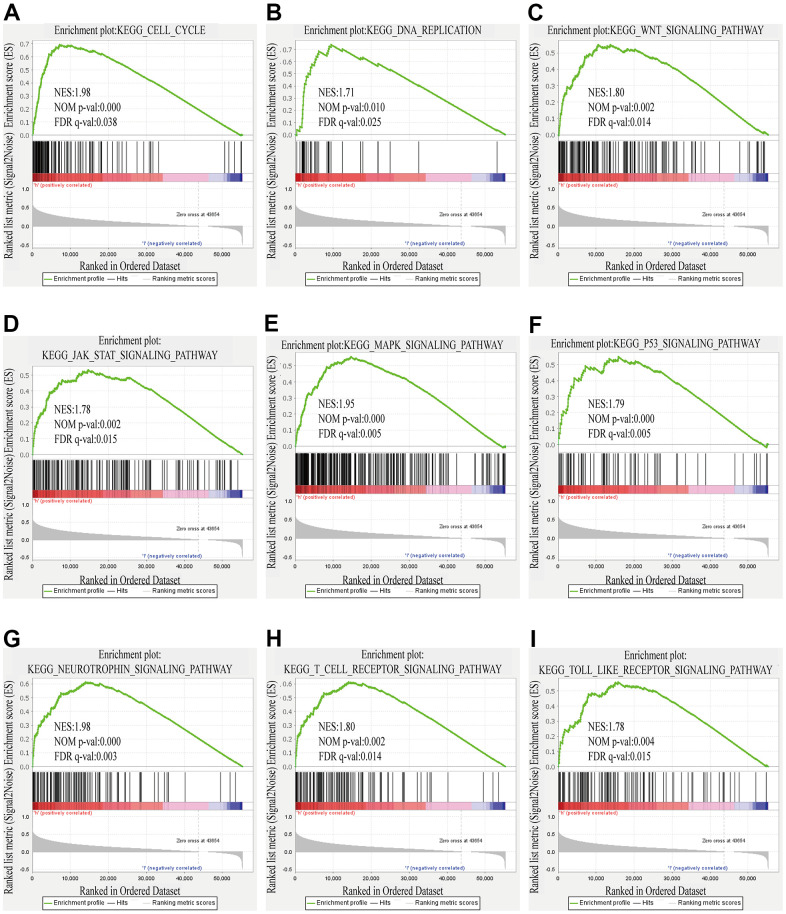
**GSEA results showed differential enrichment of genes with high ZSCAN20 expression.** (**A**) Cell cycle. (**B**) DNA replication. (**C**) WNT signaling pathway. (**D**) JAK STAT signaling pathway. (**E**) MAPK signaling pathway. (**F**) P53 signaling pathway. (**G**) Neurotrophin signaling pathway. (**H**) T cell receptor signaling pathway. (**I**) Toll like receptor signaling pathway. NES, normalized enrichment score; ES, enrichment score; FDR, false discovery rate.

In contemplation of seeking the possible biological pathways of ZSCAN20 regulation in HCC, we selected the high and low ZSCAN20 expression groups to perform GSEA analysis. Cell cycle pathways, like cell cycle, Wnt signaling pathway; some popular cancer pathways, such as MAPK signaling pathway; immune-related pathways, like T cell receptor signaling pathways, are very abundant (Supplementary Table 2) (Nom p < 0.05; FDR < 0.05; NES > 1.7). These results indicate that the ZSCAN20 expression network acts a vital part on the immunity and cell cycle mechanism of HCC (Figure 6A–6I).

### Identification of key ZSCAN20-interacting genes related to cell cycle

**Figure 7:**
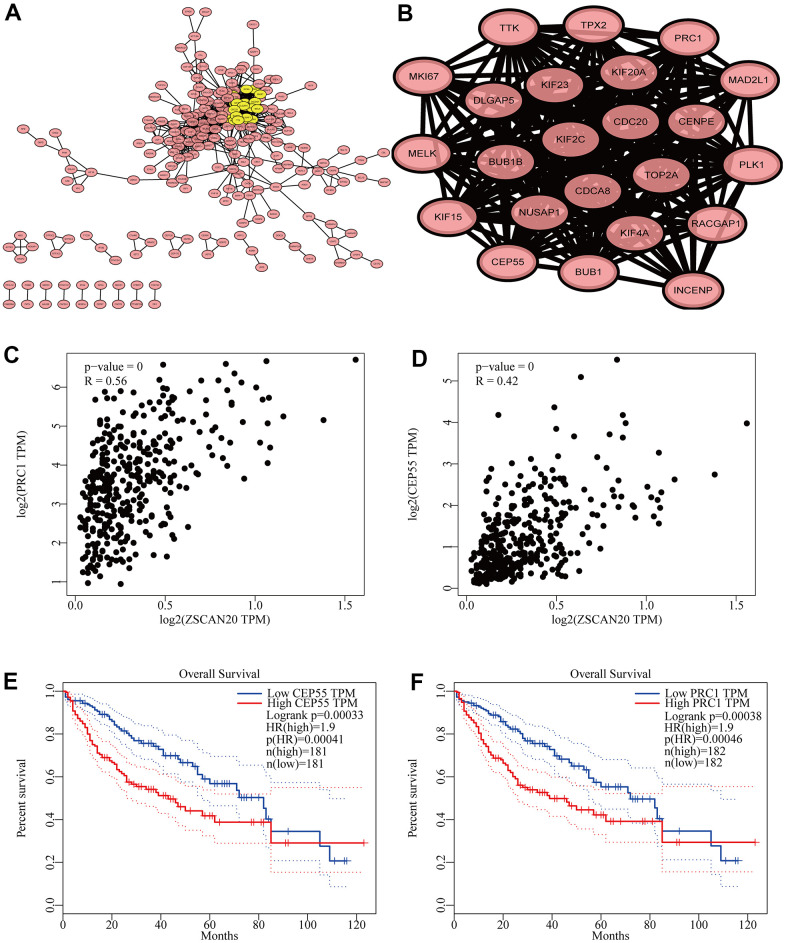
**Cell cycle related genes correlation analysis.** (**A**) Top 500 genes in LinkedOmics were filtered into protein–protein interaction network complex. (**B**) The PPI network of ZSCAN20 was generated using STRING. (**C**, **D**) Draw a scatter plot to show the correlation between the ZSCAN20 and the highest score genes (PRC1 and CEP55). (**E**, **F**) Associations with overall survival and the expression of PRC1 and CEP55.

To deeply study the hidden biological functions of ZSCAN20, we pricked off the first 500 genes in LinkedOmics through the STRING database, and then built up the PPI network of ZSCAN20 (Figure 7A). In addition, we used Cytoscape’s MCODE plug-in to build the most important modules. Then, according to k-core = 2, we calculated their connection degree and the highest average connection score (21.091) to identify the most relevant 23 central genes (Figure 7B). In addition, Supplementary Table 3 details central genes. This result initially screened 23 genes closely related to ZSCAN20. We subsequently studied these 23 genes and found that the above-mentioned genes are closely related to the cell cycle. Then, we further produced scatter plots and survival curves of ZSCAN20 and the highest-scoring genes (PRC1 and CEP55) through GEPIA. The outcomes revealed that ZSCAN20 was truly connected with PRC1 and CEP55, and the high expression of PRC1 and CEP55 led to a poor prognosis (Figure 7C–7F). The above results suggest that ZSCAN20 may impact on adjusting the cell cycle.

### ZSCAN20 knockdown inhibits HCC cell invasion, migration and proliferation

**Figure 8:**
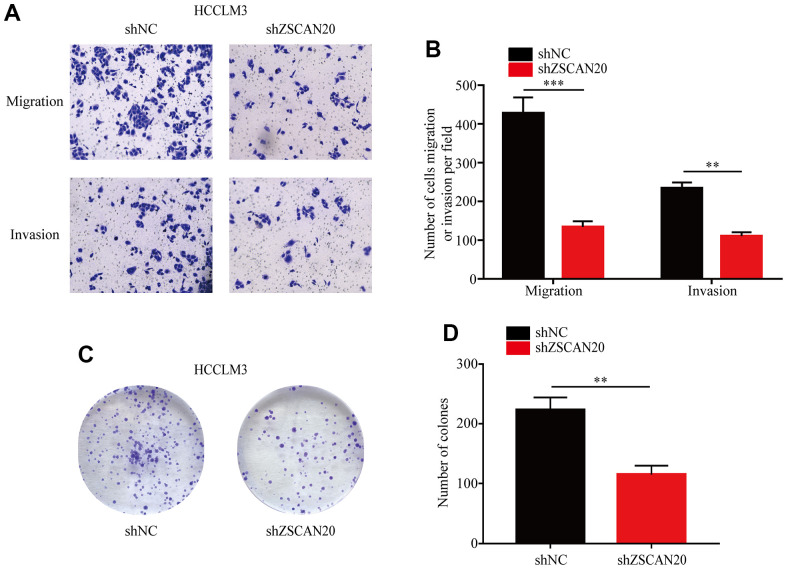
**Transwell and colony formation assays.** (**A**, **B**) Migration and invasion capacity for HCC cells treated with shZSCAN20 or shNC was detected by Transwell separately. **p < 0.01, ***p < 0.001. (**C**, **D**) Colonies formed by HCCLM3 cells transfected with control shRNA or shRNA targeting ZSCAN20. The right panels are quantification of the results of the colony formation assay.

To explore whether ZSCAN20 has a role in regulating HCC cell invasion, migration and proliferation. Invasion and migration abilities of ZSCAN20 cells were analyzed by Transwell assay to determine the role of ZSCAN20 in HCC cells. As shown, knockdown of ZSCAN20 significantly reduced the invasive and migratory abilities of HCC cells (Figure 8A, 8B). Based on colony formation assay, knockdown of ZSCAN20 strongly lowered the proliferation ability of HCC cells (Figure 8C, 8D). Collectively, our data demonstrate that ZSCAN20 knockdown significantly inhibit invasion, migration and proliferation abilities of HCC cell.

### The association of ZSCAN20 and immune infiltration in HCC

**Figure 9:**
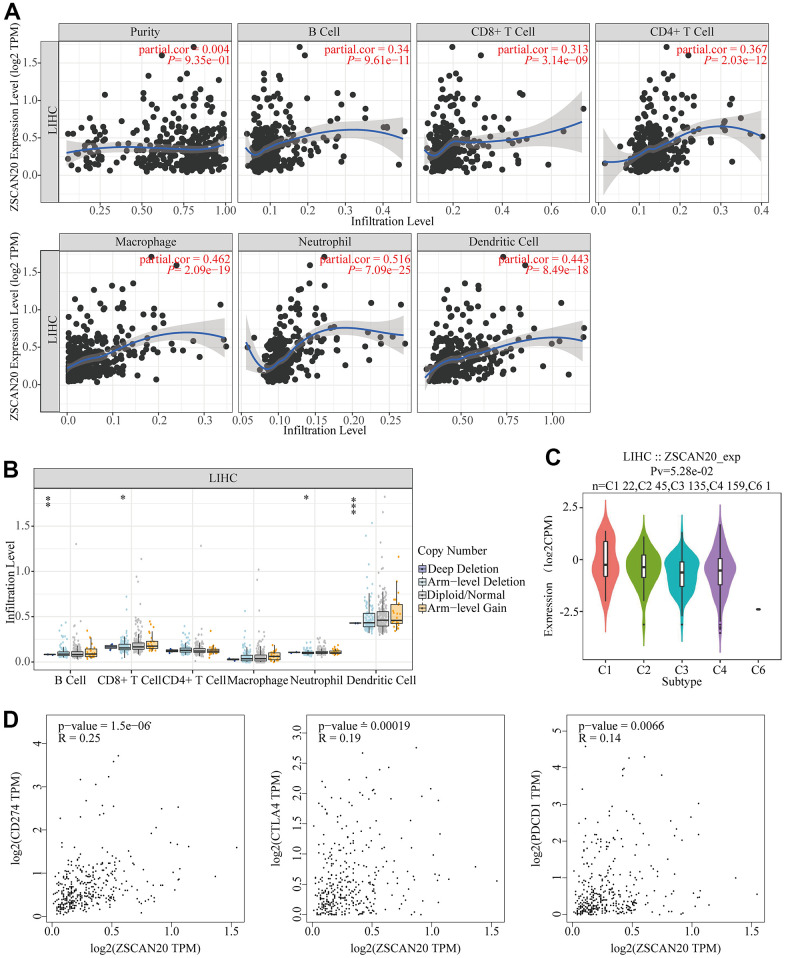
**Association between ZSCAN20 and immune cell infiltration in HCC.** (**A**) ZSCAN20 showed a significant correlation with the infiltrating abundance of B cell, CD8+ T cell, CD4+ T cell, Macrophage, Neutrophil, and Dendritic cell using the TIMER database. (**B**) The relationship between ZSCAN20 CNV and immune infiltration.*p < 0.05, **p < 0.01, ***p < 0.00. (**C**) Distribution of ZSCAN20 expression across immune subtypes in LIHC (TISIDB). The different color plots represent the five immune subtypes (C1: wound healing; C2: IFN-gamma dominant; C3: inflammatory; C4: lymphocyte-depleted and C6: TGF-b dominant). (**D**) Scatter plots of the correlations between ZSCAN20 expression and CD274, CTLA4 and PDCD1 in LIHC using the GEPIA database.

In order to understand the relationship between ZSCAN20 expression and tumor infiltrating immune cells, accordingly, we appraised the nexus between ZSCAN20 and a variety of immune cells. Via TIMER database, we knew that ZSCAN20 was related to some immune-infiltrating cells, like B cell, CD8+ T cell, CD4+ T cell, macrophage, neutrophil and dendritic cell (Figure 9A). Then, ZSCAN20 CNV was utilized to clarify the hidden mechanisms associated with different immune cell infiltration. Absence and deep loss at arm level seriously disturbed the infiltration level of B cell, CD8+ T cell, neutrophil and dendritic cell in ZSCAN20. These outcomes indicated that ZSCAN20 was closely related to the infiltration of LIHC immune cells, especially the infiltration of T cell and neutrophil (Figure 9B). Then, TISIDB was utilized to evaluate the association between immune subtypes and ZSCAN20 expression. We utilized it to explore the mRNA expression of ZSCAN20 and diverse immune subgroups in HCC, and we found that the expression of ZSCAN20 was higher in the C1 (wound healing) and C2 (IFN-γ dominant) subgroups, while the expression in the C6 (TGF-β dominant) subgroup was lower (Figure 9C). We also further studied the relationship between ZSCAN20 expression in the GEPIA database and T cell checkpoints (such as CD274, CTLA4, and PDCD1). The expression of ZSCAN20 had a significant relationship with CD274 expression (R = 0.25 P = 1.5E-06), CTLA4 (R = 0.19, P = 1.9E-04) and PDCD1 (R = 0.14, P = 6.6E-03) in HCC (Figure 9D). These outcomes prove that ZSCAN20 is intimately associated with the infiltration of immune cells.

### The correlation among ZSCAN20 and immune markers, T cell immune markers in HCC

**Figure 10:**
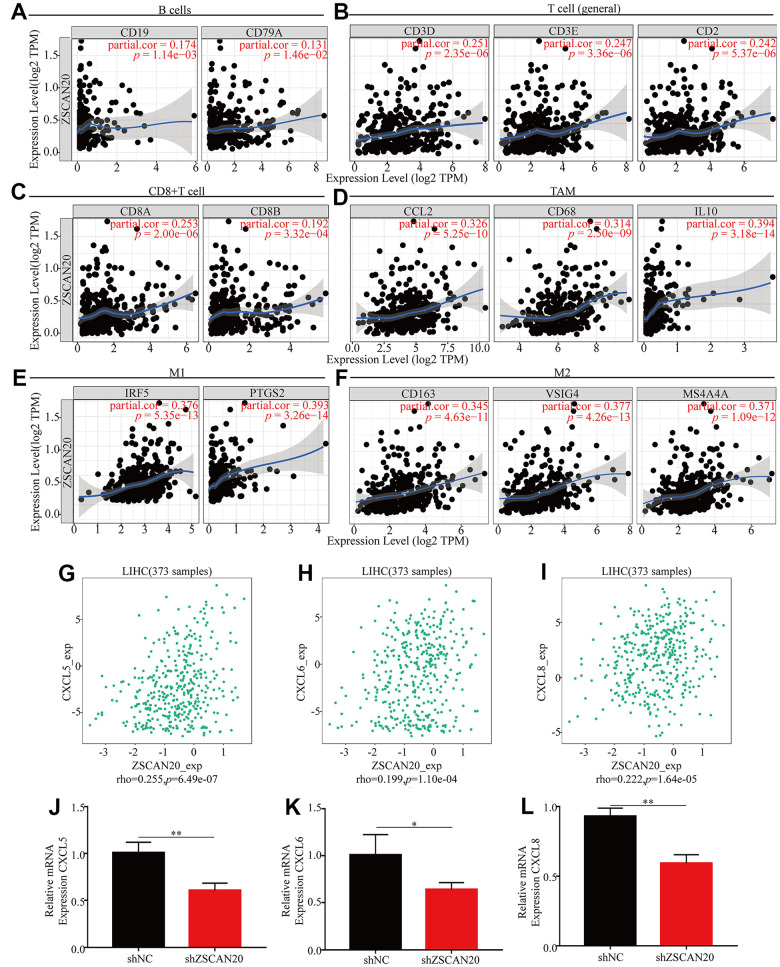
**ZSCAN20 was associated with immune cell infiltration in HCC.** The scatter plots showed relationship between ZSCAN20 and various gene markers of (**A**) B cell, (**B**) T cell, (**C**) CD8+T cell, (**D**) TAM, (**E**) M1 macrophage and (**F**) M2 macrophage in LIHC. (**G**–**I**) The association between ZSCAN20 and LIHC-related chemokines; (**J**–**L**) Immune-related chemokines expression in sh-ZSCAN20 HCC cells. *p < 0.05, **p < 0.01, ***p < 0.001.

With the purpose of going deep on ZSCAN20 expression related to different markers of multiple immune cells. We made use of the TIMER database to further study the relation, and the genes listed in Table 2 and Figure 10A–10F were used to characterize different immune cells. Tumor purity is a vital factor affecting clinical tumor biopsy immune infiltration anatomy. The expression of ZSCAN20 had a considerable positive correlation with the levels of most markers in distinguished kinds of LIHC immune cells after regulating tumor purity. In addition, we evaluated the link between ZSCAN20 and multiple T cell immune markers. We found that ZSCAN20 expression level was remarkably associated with 25 of the 26 T cell markers in LIHC after adjusting for tumor purity (Table 3). Moreover, ZSCAN20 was also closely linked to the LIHC-related chemokines including CXCL5, CXCL6 and CXCL8 (Figure 10G–10I). Besides, we did the immune-related chemokines expression analysis in HCC cells. CXCL5, CXCL6 and CXCL8 were all down-regulated in ZSCAN20 shNC cells (Figure 10J–10L). It could be known from the results that ZSCAN20 was positively associated with these chemokines.

**Table 2:** Relationship between ZSCAN20 and gene marker sets of different immune cells using the TIMER database.

**Description**	**Gene markers**	**LIHC**			
		**None**		**Purity**	
		**Cor**	**p**	**Cor**	**p**
**B cell**	CD19	0.18	**5.65E-04**	0.18	**4.30E-04**
	CD79A	0.13	**1.55E-02**	0.13	**1.46E-02**
**T cell (general)**	CD3D	0.21	**4.43E-05**	0.25	**2.35E-06**
	CD3E	0.20	**1.37E-04**	0.25	**3.36E-06**
	CD2	-0.16	**1.51E-01**	0.24	**5.37E-06**
**CD8+ T cell**	CD8A	0.22	**1.70E-05**	0.25	**2.00E-06**
	CD8B	0.17	**1.13E-03**	0.19	**3.32E-04**
**Monocyte**	CD86	0.39	**8.92E-15**	0.46	**2.89E-19**
	CSF1R	0.34	**1.09E-11**	0.40	**5.32E-15**
**TAM**	CCL2	0.29	**2.00E-08**	0.33	**5.25E-10**
	CD68	0.29	**1.52E-08**	0.31	**2.50E-09**
	IL10	0.35	**2.34E-12**	0.39	**3.18E-14**
**M1**	IRF5	0.39	**1.27E-14**	0.38	**5.35E-13**
	PTGS2	0.33	**6.93E-11**	0.39	**3.26E-14**
**M2**	CD163	0.30	**3.40E-09**	0.34	**4.63E-11**
	VSIG4	0.33	**6.25E-11**	0.38	**4.26E-13**
	MS4A4A	0.31	**8.00E-10**	0.37	**1.09E-12**
**Neutrophils**	CEACAM8	0.05	3.01E-01	0.07	1.95E-01
	ITGAM	0.52	**1.51E-27**	0.57	**1.13E-30**
	CCR7	0.20	**1.36E-04**	0.23	**1.82E-05**
**Natural killer cell**	KIR2DL1	0.04	4.21E-01	0.03	5.80E-01
	KIR2DL3	0.19	**2.50E-04**	0.20	**1.26E-04**
	KIR2DL4	0.19	**2.81E-04**	0.19	**3.08E-04**
	KIR3DL1	0.12	**2.67E-02**	0.12	**2.63E-02**
	KIR3DL2	0.07	1.55E-01	0.09	8.17E-02
	KIR3DL3	0.04	4.89E-01	0.03	6.19E-01
**Dendritic cell**	HLA-DPB1	0.27	**7.73E-08**	0.31	**6.42E-09**
	HLA-DQB1	0.20	**9.16E-05**	0.24	**9.28E-06**
	HLA-DRA	0.32	**1.50E-10**	0.36	**3.64E-12**
	HLA-DPA1	0.31	**7.28E-10**	0.36	**7.17E-12**
	CD1C	0.14	**6.97E-03**	0.15	**6.06E-03**
	NRP1	0.45	**2.55E-20**	0.45	**9.17E-19**
	ITGAX	0.40	**6.13E-16**	0.46	**1.29E-19**

**Table 3:** Relationship between ZSCAN20 and gene marker sets of different T cells using the TIMER database.

**Description**	**Gene markers**	**LIHC**			
		**None**		**Purity**	
		**Cor**	**p**	**Cor**	**p**
Th1 cell	TBX21	0.15	**2.90E-03**	0.18	**6.32E-04**
	STAT4	0.23	**5.63E-06**	0.26	**9.94E-07**
	STAT1	0.41	**2.76E-16**	0.42	**5.11E-16**
	TNF	0.35	**6.32E-12**	0.39	**5.29E-14**
	HAVCR2	0.42	**4.77E-17**	0.50	**8.99E-23**
Th1-like cell	CXCR3	0.20	**9.27E-05**	0.23	**2.14E-05**
	BHLHE40	0.45	**9.79E-20**	0.45	**9.69E-19**
	CD4	0.30	**3.31E-09**	0.32	**7.80E-10**
Th2 cell	STAT6	0.31	**1.13E-09**	0.30	**2.04E-08**
	STAT5A	0.38	**1.61E-14**	0.39	**9.88E-14**
	FOXP3	0.24	**5.78E-16**	0.26	**2.22E-17**
Treg cell	CCR8	0.45	**1.40E-38**	0.49	**7.97E-44**
	TGFB1	0.37	**1.58E-13**	0.40	**1.31E-14**
Resting Treg cell	FOXP3	0.24	**5.78E-16**	0.26	**2.22E-17**
	IL2RA	0.38	**1.98E-14**	0.44	**1.21E-17**
	FOXP3	0.24	**5.78E-16**	0.26	**2.22E-17**
Effector Treg cell	CCR8	0.45	**1.40E-38**	0.49	**7.97E-44**
	TNFRSF9	0.41	**2.11E-16**	0.45	**2.19E-18**
	CX3CR1	0.40	**6.37E-16**	0.41	**8.63E-16**
Effector T cell	FGFBP2	-0.06	2.60E-01	-0.05	4.02E-01
	FCGR3A	0.42	**4.40E-17**	0.46	**2.33E-19**
	CCR7	0.20	**6.47E-08**	0.23	**1.23E-09**
Naive T cell	SELL	0.31	**1.95E-18**	0.35	**2.29E-21**
Effector memory T cell	DUSP4	0.39	**1.43E-14**	0.43	**2.94E-17**
	GZMK	0.11	**2.78E-02**	0.14	**8.94E-03**
	GZMA	0.14	**6.21E-03**	0.17	**1.34E-03**
Resident memory T cell	CD69	0.25	**1.17E-06**	0.30	**1.37E-08**
	CXCR6	0.20	**7.26E-05**	0.26	**1.33E-06**
	MYADM	0.44	**2.79E-19**	0.45	**2.86E-18**
General memory	CCR7	0.20	**6.47E-08**	0.23	**1.23E-09**
	SELL	0.31	**1.95E-18**	0.35	**2.29E-21**

**Figure 11:**
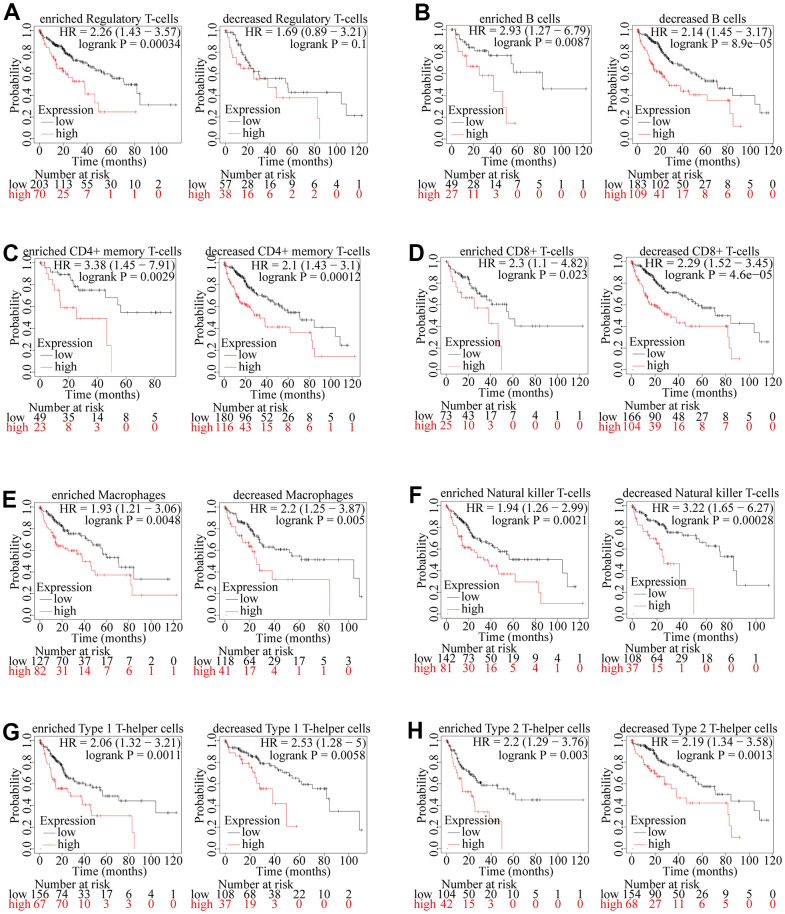
**Kaplan-Meier survival curves according to high and low expression of ZSCAN20 in immune cell subgroups in HCC.** (**A**–**H**) Correlations between ZSCAN20 expression and OS in different immune cell subgroups in HCC patients were estimated by Kaplan-Meier plotter.

According to the results above, we have confirmed that the expression of ZSCAN20 was positively correlated with the infiltration of most immune cells in HCC, and the expression of ZSCAN20 was also associated with the poor prognosis of these tumors. Therefore, we speculated that part of the reason that ZSCAN20 expression affected the poor prognosis of HCC is immune infiltration. We performed a prognostic analysis of ZSCAN20 expression of patients in different immune cell subsets by Kaplan-Meier Plotter. We found that in enriched regulatory T cell cohorts, high expression of ZSCAN20 led to poor prognosis in HCC patients, but there was no significant association between low/high ZSCAN20 expression and HCC patient prognosis in decreased Regulatory T-cells cohorts (Figure 11A). Nonetheless, in other both enriched and decreased immune cell groups, it revealed that high expression of ZSCAN20 resulted in poor prognosis (Figure 11B–11H). These results supported our speculation that high ZSCAN20 expression levels all contributed to the poor prognosis of HCC patients in part due to the level of regulatory T-cell immune infiltration. It is these consequences that show ZSCAN20 has a close association with the infiltration of HCC immune infiltrating cells, and may be associated with immune infiltration of regulatory T cells.

### Correlation between ZSCAN20 expression and m6A modification in HCC

**Figure 12:**
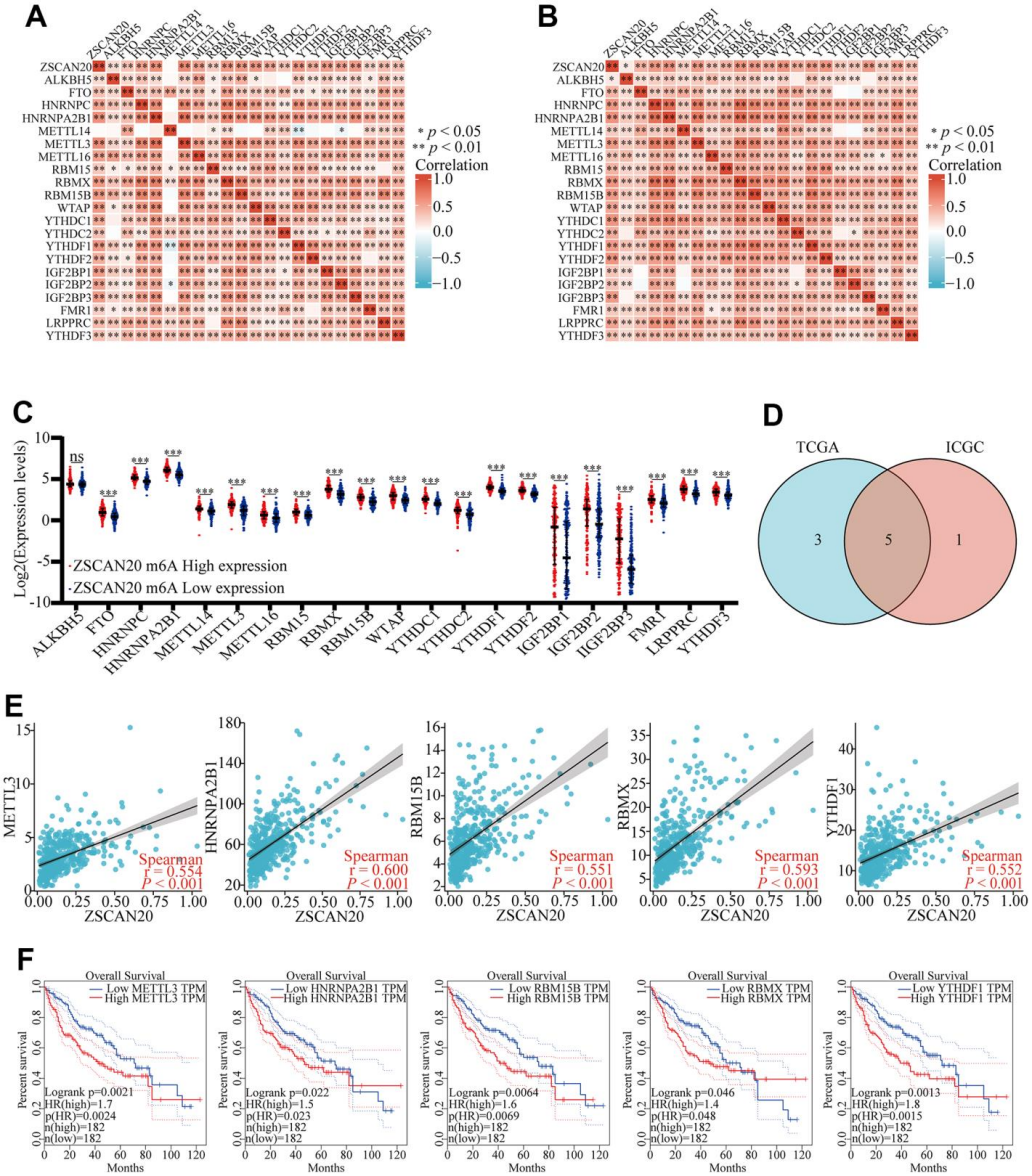
**Correlations of ZSCAN20 expression with m6A related genes in HCC.** (**A**, **B**) TCGA LIHC and ICGC data sets were used to analyze the correlation between the ZSCAN20 and the m6A related genes expression in LIHC. (**C**) Draw a scatter plot to show the correlation between the ZSCAN20 and the m6A related genes expression, including METTL3, HNRNPA2B1, RBM15B, RBMX and YTHDF1. (**D**) The differential expression of m6A related genes between high and low ZSCNA20 expression groups in LIHC tumor samples. (**E**) Venn diagram showed both expression correlation and differential expression of genes. (**F**) Kaplan-Meier curve of METTL3, HNRNPA2B1, RBM15B, RBMX, YTHDF1. *p < 0.05, **p < 0.01, ***p < 0.001, ****p < 0.0001. ns, not significant.

Exploring whether ZSCAN20 can be modified by m6A played a significant role in the growth of HCC. We obtained the TCGA and ICGC data sets to research the relation between ZSCAN20 expression and the expression of 21 m6a-related genes in LIHC. The outcomes showed that the expression of ZSCAN20 had a positive relationship with many m6a-related genes (Figure 12A, 12B) (P < 0.05). Meanwhile, With the purpose of ascertaining whether there was a difference between high and low expression of m6A modification expressed by ZSCNA20 in LIHC, we tried to constrain the differential expression of m6A-related genes between high and low ZSCAN20 expression groups, and it was the outcomes that showed ZSCAN20 high expression groups were higher than those in the low expression groups (P < 0.05) (Figure 12C). We selected genes to draw a Venn diagram (correlation coefficient of TCGA and ICGC > 0.55), which showed the correlation and differential expression of genes, including METL3, HNRNPA2B1, RBM15B, RBMX and YTHDF1 (Figure 12D). Then, we produced scatter plots of ZSCAN20 and m6A related genes, which showed that ZSCAN20 was momentously positively associated with METL3, HNRNPA2B1, RBM15B, RBMX and YTHDF1 (Figure 12E). We further used GEPIA to study the prognosis of related genes, and the outcomes of GEPIA displayed the high expression of METL3, HNRNPA2B1, RBM15B, RBMX and YTHDF1 were closely related to the poor prognosis of LIHC (P < 0.05) (Figure 12F). These results indicate that ZSCAN20 may have an intimate relationship with the m6A modification of LIHC, may affecting the occurrence and development of tumors together with m6A-related genes.

## DISCUSSION

As the fifth most common cancer globally, HCC is a malignant tumor with high mortality. Patients with HCC often lack evident clinical symptoms in the first instance. Despite the discovery and treatment, patients may still have tumor progression or recurrence. In order to study new tumor treatment methods, such as the use of checkpoint inhibitors for immunotherapy [41–43] except for AFP [44], new biomarkers have not yet entered daily practice. Therefore, research to find a new and meaningful biomarker to assist HCC detection and treatment is imminent. Here we applied various bioinformatics analysis for the purpose of investigating ZSCAN20’s expression and prognosis in HCC. At the same time, study its correlation with cell cycle, immune infiltration and m6A.

First, we found that ZSCAN20 was highly expressed in HCC through public databases and HCC clinical disease samples. The UALCAN results suggested that ZSCAN20 was closely connected to the clinicopathological features. The Kaplan-Meier Plotter survival analysis showed high ZSCAN20 expression was linked to low survival rate. And Cox analysis suggested that ZSCAN20 can be viewed as an independent predictor. Studies have shown that epigenetic changes may increase the risk of cancer by altering the expression of oncogenes [45]. DNA methylation is one type of epigenetic change that can be considered as a potential marker of cancer development and progression [46]. In HCC, according to our results, ZSCAN20 was hypermethylated in HCC. Meanwhile, the hypermethylation of ZSCAN20 was also associated with low survival rates. These results indicated that in HCC, ZSCAN20 was highly expressed and also hypermethylated. Both its high expression and hypermethylation indicated a poor prognosis.

In order to confirm our hypothesis, we further studied the biological function of ZSCAN20 in HCC. In order to analyze the biological pathways that ZSCAN20 can regulate in HCC, we used LinkedOmics to investigate the biological functions of ZSCAN20 through co-expressed genes. GO and KEGG results suggested that ZSCAN20 was mainly associated with cell cycle, DNA replication and immune infiltration. In addition, GSEA results showed that ZSCAN20 was related to cancer pathways such as the famous TP53 signaling pathway, MAPK signaling pathway. At the same time, GSEA results also showed that ZSCAN20 was related to cell cycle, Wnt signaling pathway and other famous cell cycles. Wnt signaling pathway vitally participates in cell cycle regulation, physiological behavior, disease and tumorigenesis, which is related to T cell factors and lymphatic enhancement. Factors combine to form complexes which affect cell proliferation, apoptosis, and migration [47, 48]. We still need to further study whether ZSCAN20 affected the occurrence and development of cancer over Wnt signaling pathway in the cell cycle mechanism.

In order to further study its potential biological functions, the PPI results showed that the first 23 genes associated with ZSCAN20 were correlated with the cell cycle. It was well known that the anomalous expression of cell cycle related proteins may affect cell growth and chromosomal instability, causing cancer progression. We selected the two co-expressed genes (PRC1 and CEP55) with the highest cluster scores and found that they had a strong correlation with ZSCAN20. Subsequently, we conducted survival analysis of these two genes, and the results indicated their high expression can cause poor prognosis. Studies have pointed out that the dysregulation of the protein regulator 1 (PRC1) of cytokinesis led to cytokinesis defects in the cell cycle, which in turn promoted chromosomal instability (CIN), thereby promoting tumor heterogeneity and cancer progression [49]. In addition, the absence of CEP55 leads to mitotic arrest and mitotic cell death in the cell cycle, and CEP55 has been proven to be an anti-mitotic drug targeting the mitotic mechanism of the cell cycle to eliminate cancer cells [50]. This implied that ZSCAN20 may get involved in HCC through its association with cell cycle-related signal pathways.

The above studies confirmed that ZSCAN20 was highly expressed in HCC and was closely related to poor prognosis. At the same time, the abilities of invasion, migration and proliferation are the main factors to evaluate the progression of malignant tumor [51, 52]. To investigate the effect of ZSCAN20 on malignant pathological processes in HCC. Through Transwell and colony formation assays, we found that interfering with the expression of ZSCAN20 could significantly inhibit the invasion, migration and proliferation abilities of HCC cells.

As one of the most major organs, the liver has a variety of immune functions. HCC is closely associated with inflammation, and immune tolerance and escape have an important impact on inflammation [53]. Most cancers develop immune tolerance and escape by impairing the function of immune cells or releasing inhibitory cytokines [54]. Recent studies have shown that tumor infiltrating immune cells (TIIC) have a key influence regulating and controlling tumor progression. Moreover, TIICs are preferentially enriched in HCC and may be cloned and expanded [55], and the accumulation of TIICs is associated with poor prognosis of HCC [56]. Functional network analysis results showed the biological function of ZSCAN20 in HCC was directly associated with the immune response. Thus, we explored whether the expression of ZSCAN20 can affect HCC immune cells in the TIMER database. We found ZSCAN20 expression was extremely related to tumor purity. Among the six immune cells, the level of ZSCAN20 was positively correlated with the infiltration of CD8+T cells, CD4+T cells, macrophages, neutrophils and dendritic cells. Significant associations between ZSCAN20 and diverse immune cell marker sets were discovered in HCC. Studies have reported that insufficient crosstalk between dendritic cells (DC) and T cells can cause HCC tumor tolerance [57]. In HCC patients, Treg binds to lots of activated CD8+ cytotoxic cells and is related to OS and DFS [58]. Furthermore, Tregs have been found to have profound roles in regulating immune responses and maintaining self-tolerance and immune homeostasis. While Tregs play an important role in preventing autoimmune diseases, Tregs can regulate the adaptive immune system by regulating CD4 Th cells, CD8 T cells and B cells. At the same time, Tregs also regulate innate immune system cells (including dendritic cells, macrophages, neutrophils, γδ T cells, natural killer and innate lymphoid cells) [59]. The study also found that Tregs also played an important role in HCC [60]. Here ZSCAN20 was closely related to various subtypes of T cells, including memory T, effector T, Th1-like, effector Treg and exhausted T cell. Meanwhile, the high expression of ZSCAN20 was correlated with poor prognosis in the enriched Regulatory T-cell. Recently, Immune Checkpoint Inhibitor (ICI) is a swiftly developing immunotherapy method for liver cancer [61]. ICI can activate tumor-specific T cells to fight tumors [62]. Here we found that ZSCAN20 was significantly related to T cell checkpoints (CD274, CTLA4 and PDCD1). Additionally, it was found through TISIDB that ZSCAN20 was positively correlated with CXCL5, CXCL6 and CXCL8, and interference with ZSCAN20 can significantly reduce the expression of these chemokines. Our findings suggested that in HCC ZSCAN20 may exert biological functions through immune cells, and ZSCAN20 may regulate the development and prognosis of HCC through Regulatory T-cell.

Methylation occurs at the N position of the sixth adenylate, that is, N6-methyladenosine, referred to as m6A [63], which is common and numerous post-transcriptional modification on mRNA and lncRNA [64]. M6A is used in various biological processes and diseases. Disease pathogenesis and tumorigenesis and development process play a pivotal role [65]. Studies have shown that HNRNPA2B1, as an m6A reader, mediates alternative splicing of target RNA and enhances primary miRNA processing [66], which is highly expressed in liver cancer [67]. RBMX is a m6A regulatory factor [68], that is upregulated in HCC, and RBMX expression is positively related to the viability and proliferation of HCC cells, promoting the development of HCC and sorafenib resistance [69]. The results of our study showed that there was a positive correlation between ZSCAN20 and the expression of various m6A-related genes in TCGA and ICGC, and it was found that METL3, HNRNPA2B1, RBM15B, RBMX and YTHDF1 have the most obvious relationship with ZSCAN20. Further research we also found that when ZSCAN20 was highly expressed and lowly expressed, and most of the m6A-related genes showed differential expression. Also, by constructing a Kaplan-Meier curve, it was found that the high expression group of METTL3, HNRNPA2B1, RBM15B, RBMX and YTHDF1 had a shorter survival time. In summary, our research results indicated that the mechanism of ZSCAN20’s role in HCC may be related to m6A modification. ZSCAN20 may affect HCC mRNA methylation level through its association with METTL3, HNRNPA2B1, RBM15B, RBMX, YTHDF1, and ultimately affected the progress of HCC.

This study has some limitations. Firstly, the data in the database is constantly updated and may affect the results obtained on the results website. We need to collect more clinical data for verification. Secondly, our study only focused on HCC and normal tissues. Jiang et al. found that Hsa_circ_0028502 and hsa_circ_0076251 distinguish HCC tissues, liver cirrhosis (LC) and chronic hepatitis (CH) [70]. We can collect more clinical data to further explore the development of HCC, analyzing the expression of ZSCAN20 in HCC tissues, LC tissues and CH tissues and whether ZSCAN20 can be used as a biomarker to distinguish HCC tissues, LC tissues and CH tissues. Finally, Tian et al. found the prognostic value of TPM1–4 as a risk model in HCC [71]. Although ZSCAN20 can be used as an independent prognostic factor for HCC, we can also study other genes in ZSCAN20’s family and combine them into a risk model in future studies. The accuracy of prediction may be greatly improved, and it may also be able to predict different types of HCC patients.

On a final note, using bioinformatics methods as well as experimental verification, this study has proved ZSCAN20 is upregulated in HCC. ZSCAN20 expression is correlated with the clinicopathological characteristics and prognosis of HCC patients. In addition, ZSCAN20 may have an influence on HCC through cell cycle and immune infiltration, m6A modification and other processes. Interference with ZSCAN20 could inhibit invasion, migration and proliferation abilities of HCC cell. Collectively, ZSCAN20 can be used as a biomarker for the diagnosis, treatment and prognosis of HCC.

## Supplementary Material

Supplementary Tables
