# Correction for: Comprehensive analysis of prognostic value, relationship to cell cycle, immune infiltration and m6A modification of ZSCAN20 in hepatocellular carcinoma

**DOI:** 10.18632/aging.204699

**Published:** 2023-04-29

**Authors:** Fang Jiayu, Yike Jiang, Xuanrui Zhou, Minqin Zhou, Jingying Pan, Yun Ke, Jing Zhen, Da Huang, Weifan Jiang

**Affiliations:** 1 Second Affiliated Hospital of Nanchang University, Nanchang, China; 2 Second College of Clinical Medicine, Nanchang University, Nanchang, China; 3 Department of Thyroid Surgery, Second Affiliated Hospital of Nanchang University, Nanchang, China; 4 Department of Urology, Second Affiliated Hospital of Nanchang University, Nanchang, China

**Keywords:** hepatocellular carcinoma, ZSCAN20, biomarker, prognosis, immune infiltrates

**This article has been corrected:** The authors updated the text of the **Quantitative real-time PCR** section with the sequences of the real-time PCR primers.

Corrected **Materials and methods** section is presented below.

## Quantitative real-time PCR

Real-time PCR was performed on fresh frozen samples. Total RNA was isolated from tissues using the TRIzol reagent (Thermo Fisher Scientific). Then, according to standard methods, qRTPCR analysis was performed to observe the expression level of ZSCAN20. Primers for ZSCAN20 from 5’ to 3’: TGGCCTGGTCAATGTTGAGT (F), TGCACACCTGCAATACGACT (R). Primers for β-actin from 5’ to 3’: TGGAACGGTGAAGGTGACAG (F), AACAACGCATCTCATATTTGGAA (R).

