# Supplementary material for: Comprehensive analysis of prognostic value, relationship to cell cycle, immune infiltration and m6A modification of ZSCAN20 in hepatocellular carcinoma
Source: Aging (Albany NY). 2022 Dec 3;14(23):9550–78. doi: 10.18632/aging.204312 (PMC9792207; doi:10.18632/aging.204312)
Supplement: Supplementary Tables [file aging-14-204312-s001.pdf]

SUPPLEMENTARY TABLES

Supplementary Table 1. Logistic analysis of the association between ZSCAN20 expression and clinical characteristics.

Clinical characteristics	Total (N)	0dds ratio in ZSCAN20 expression	P value
**Age** (> 60 vs ≤ 60)	376	1.11(0.74-1.68)	0.603
**Gender** (Female vs. Male)	377	1.32(0.86-2.05)	0.210
**grade**(III vs I)	372	2.92(1.51-5.76)	**0.002**
**Stage** (II vs I)	353	1.82(1.08-3.09)	**0.025**
**T** (T2 vs T1)	374	1.86(1.13-3.10)	**0.016**
**N** (N1 vs N0)	261	3.05(0.38-62.07)	0.337

T, tumor; N, node; M, metastasis; Bold values indicate P-values < 0.05.

Supplementary Table 2. Gene sets enriched in the high ZSCAN20 expression phenotype.

Gene set name	NES	NOM p-val	FDR q-val
KEGG_CELL_CYCLE	1.98	0	0.004
KEGG_DNA_REPLICATION	1.71	0.01	0.025
KEGG_WNT_SIGNALING_PATHWAY	1.8	0.002	0.014
KEGG_JAK_STAT_SIGNALING_PATHWAY	1.78	0.002	0.015
KEGG_MAPK_SIGNALING_PATHWAY	1.95	0	0.005
KEGG_P53_SIGNALING_PATHWAY	1.79	0	0.014
KEGG_NEUROTROPHIN_SIGNALING_PATHWAY	1.98	0	0.003
KEGG_T_CELL_RECEPTOR_SIGNALING_PATHWAY	1.8	0.002	0.014
KEGG_TOLL_LIKE_RECEPTOR_SIGNALING_PATHWAY	1.78	0.004	0.015

NES, normalized enrichment score; NOM, nominal; FDR, false discovery rate. Gene sets with NOM P-value <0.01 and FDR q-value <0.01 were considered as significantly enriched.

Supplementary Table 3. Characteristics of the first 23 nodes in the PPI network.

	MCODE::Clusters (1)	MCODE::Node status (1)	MCODE::Score (1)	Name	Selected	Shared name
1	Cluster 0	Seed	16.80116959	CEP55	FALSE	CEP55
2	Cluster 0	Clustered	16.80116959	PRC1	FALSE	PRC1
3	Cluster 0	Clustered	16.64210526	NUSAP1	FALSE	NUSAP1
4	Cluster 0	Clustered	16.64210526	KIF20A	FALSE	KIF20A
5	Cluster 0	Clustered	16.64210526	PLK1	FALSE	PLK1
6	Cluster 0	Clustered	16.64210526	BUB1	FALSE	BUB1
7	Cluster 0	Clustered	16.64210526	BUB1B	FALSE	BUB1B
8	Cluster 0	Clustered	16.64210526	TOP2A	FALSE	TOP2A
9	Cluster 0	Clustered	16.64210526	KIF4A	FALSE	KIF4A
10	Cluster 0	Clustered	16.64210526	TPX2	FALSE	TPX2
11	Cluster 0	Clustered	16.64210526	CDCA8	FALSE	CDCA8
12	Cluster 0	Clustered	16.64210526	KIF23	FALSE	KIF23
13	Cluster 0	Clustered	16.64210526	DLGAP5	FALSE	DLGAP5
14	Cluster 0	Clustered	16.64210526	KIF2C	FALSE	KIF2C
15	Cluster 0	Clustered	16.64210526	CENPE	FALSE	CENPE
16	Cluster 0	Clustered	16.64210526	CDC20	FALSE	CDC20
17	Cluster 0	Clustered	15.89542484	KIF15	FALSE	KIF15
18	Cluster 0	Clustered	15.89542484	MAD2L1	FALSE	MAD2L1
19	Cluster 0	Clustered	15.71929825	RACGAP1	FALSE	RACGAP1
20	Cluster 0	Clustered	15.16190476	TTK	FALSE	TTK
21	Cluster 0	Clustered	15	MKI67	FALSE	MKI67
22	Cluster 0	Clustered	14.77941176	MELK	FALSE	MELK
23	Cluster 0	Clustered	14	INCENP	FALSE	INCENP
